# Breaking the Toughness‐Stretchability Trade‐Off in Hydrogels with Dynamic Hydrogen Bonding

**DOI:** 10.1002/advs.202522530

**Published:** 2026-03-14

**Authors:** Yining Gao, Yong Tao, Roland J.‐M. Pellenq, Fazhou Wang

**Affiliations:** ^1^ State Key Laboratory of Silicate Materials for Architectures Wuhan University of Technology Wuhan China; ^2^ Department of Civil and Environmental Engineering & Research Centre for Resources Engineering Towards Carbon Neutrality The Hong Kong Polytechnic University Hong Kong China; ^3^ Institut Européen des Membranes CNRS and Université of Montpellier Montpellier France

**Keywords:** hybrid phyllosilicate, hydrogen bonding, hydrogel, polyacrylamide

## Abstract

The inherent trade‐off between toughness and stretchability in conventional hydrogels restricts their utility in demanding structural and engineering scenarios. Here, we address this limitation by designing a nanocomposite hydrogel with densified, interfacially bridged network architecture, comprising uniformly dispersed aminopropyl‐hybrid‐phyllosilicate (AHPS) nanosheets within a polyacrylamide (PAM) matrix. A dynamic hydrogen‐bonding network between AHPS and PAM enables efficient energy dissipation during deformation, imparting the material with exceptional mechanical performance. The optimized nanocomposite (3 wt.% AHPS) achieves a toughness of 6.91 MJ/m^3^—a 173‐fold enhancement compared to pristine PAM—and an elongation at break of 3390%, representing a 31‐fold improvement over the unreinforced hydrogel. Furthermore, the reversible breakage and reformation of hydrogen bonds endow the AHPS/PAM hydrogel with outstanding self‐recovery capabilities, retaining structural integrity over repeated stress‐strain cycles. By synergizing a nanoscale interfacial bridging with dynamic hydrogen bonding, this strategy unlocks unprecedented combinations of toughness, stretchability, and resilience, suggesting strong potential as a mechanically robust platform for soft robotics and flexible material systems.

## Graphical abstract

A novel nanocomposite hydrogel architecture overcomes the inherent conflict between toughness and stretchability by integrating uniformly dispersed aminopropyl‐hybrid‐phyllosilicate nanosheets within a polyacrylamide matrix. This design leverages a dynamic hydrogen‐bonding network to facilitate efficient energy dissipation and self‐recovery. The resulting material achieves exceptional mechanical performance, offering a robust platform for soft robotics and flexible engineering applications.

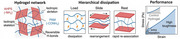

## 1 Introduction

The biocompatibility and tunable properties of hydrogels confer significant promise in diverse fields, spanning biomedicine and soft robotics [1, 2, 3]. Load‐bearing applications, however, present a critical challenge: the simultaneous requirement of high toughness and high stretchability [4, 5, 6]. This requirement arises from the fundamental trade‐off inherent in hydrogel design, where an increase in crosslinking density, while enhancing toughness, concurrently compromises stretchability, and vice versa. The reliance of existing tough hydrogels on irreversible crosslinking or high filler loadings results in a severe limitation of stretchability and fatigue resistance, precluding their use in applications involving repeated deformation, such as artificial muscles or load‐bearing implants [4, 7, 8]. The enhancement of hydrogel toughness through traditional strategies, including rigid filler incorporation or crosslinking density augmentation, inevitably leads to a compromise in stretchability [9, 10, 11]. Conversely, strategies focused on enhancing polymer chain flexibility for improved stretchability often result in diminished toughness [12, 13]. The circumvention of this persistent limitation necessitates the development of innovative materials design strategies capable of both effective energy dissipation and accommodation of large deformations without sacrificing structural integrity.

Incorporating dynamic interactions within the hydrogel network represents a promising approach. Unlike permanent crosslinks, dynamic bonds can rupture and reform under stress, providing a mechanism for both energy dissipation and self‐healing, thereby enabling the simultaneous achievement of high toughness and stretchability [14, 15]. These dynamic interactions operate on timescales relevant to mechanical deformation (milliseconds to seconds), allowing the material to adapt to applied stress. While exploration of dynamic bonds has encompassed metal‐ligand coordination and host‐guest interactions, the unique combination of moderate bond strength and rapid dynamics offered by hydrogen bonding renders it particularly suitable for achieving the desired combination of toughness and stretchability [16, 17, 18]. However, relying solely on polymer‐polymer hydrogen bonding interactions often limits the maximum toughness that can be achieved.

In this work, we design a high‐performance nanocomposite (NC) hydrogel by strategically leveraging dynamic hydrogen bonding between aminopropyl‐hybrid‐phyllosilicate (AHPS) nanosheets and a polyacrylamide (PAM) matrix. Unlike conventional fillers like montmorillonite or graphene oxide, which rely on large platelets as physical crosslinkers to form heterogeneous networks with limited stretchability [19, 20, 21], AHPS offers a distinct advantage: its amine‐rich, tunable surface chemistry enhances aqueous dispersibility and increases the density of specific hydrogen‐bonding and weak coordination motifs with PAM. This promotes uniform dispersion and interfacial‐bridging‐driven densification with short‐range stacking under swollen conditions. The resulting network favors spatially distributed, reversible bond rupture and reformation under load, delaying damage localization and mitigating premature failure. The abundant amide groups in PAM provide an ideal platform for multipoint hydrogen bonding with AHPS, synergistically combining nanofiller reinforcement with the reversible, dissipative nature of noncovalent interactions. Through systematic investigation, we elucidate the influence of AHPS incorporation on the mechanical properties, microstructure, and dynamic interactions within AHPS/PAM NC hydrogels, establishing a clear link between material design, molecular interactions, and macroscopic performance.

## 2 Results

### 2.1 Homogeneity and Multiscale Microstructure

**FIGURE 1:**
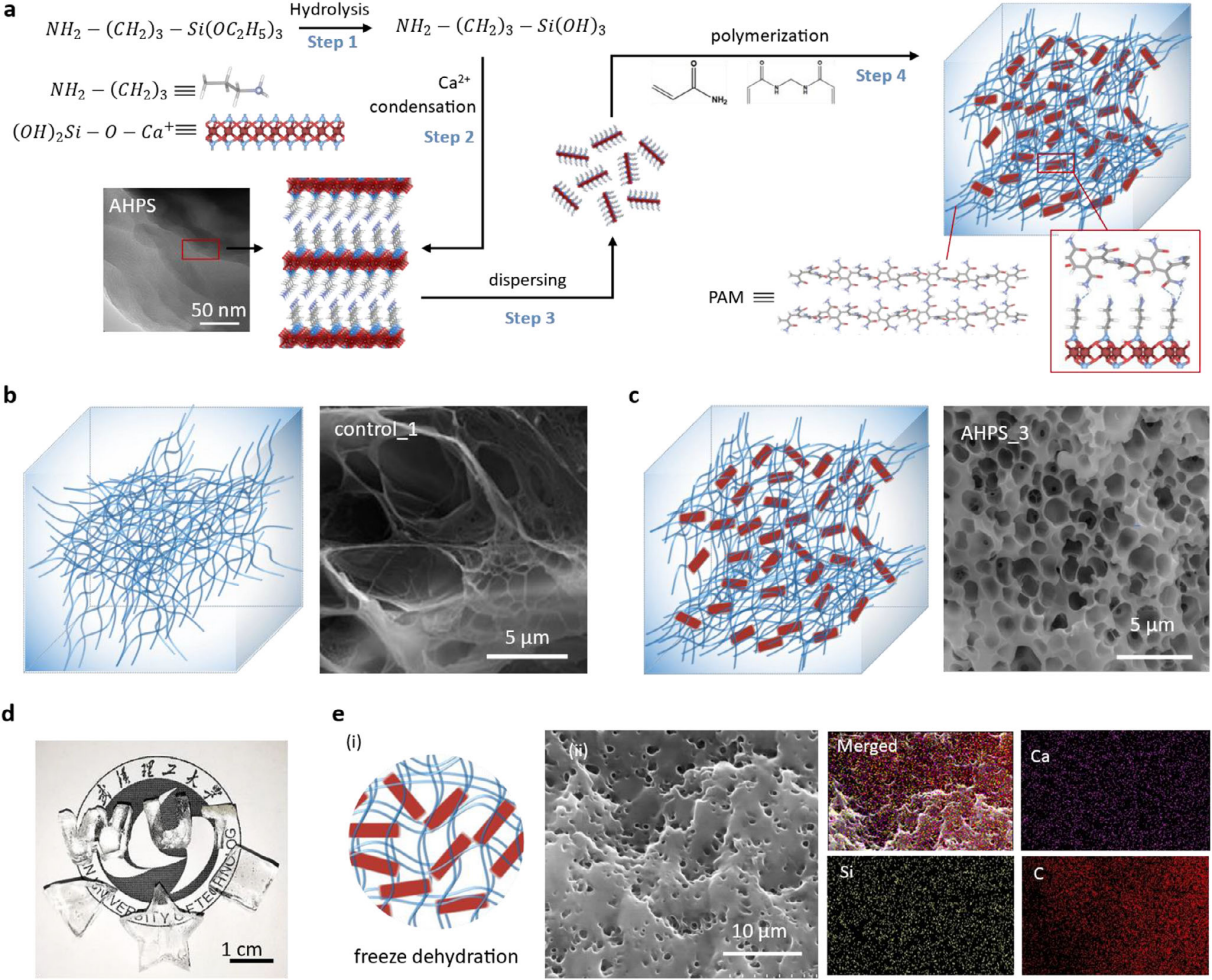
Synthetic process and structure characterization of AHPS/PAM NC hydrogels. (a) Schematic illustration of the synthetic route for AHPS/PAM NC hydrogels via in situ radical polymerization of acrylamide in the presence of AHPS nanosheets. The inset shows a transmission electron microscopy (TEM) image of AHPS, revealing its layered structure. SEM images of (b) the pure PAM hydrogel (control_1), exhibiting a disordered 3D network, and (c) the AHPS/PAM hydrogel with 3 wt.% AHPS (AHPS_3), demonstrating well‐organized cellular network. (d) Photographs of AHPS/PAM NC hydrogels, showcasing their high optical transparency and facile moldability. Wuhan University of Technology logo. Wuhan University of Technology. All rights reserved. (e) Elemental mapping of freeze‐dried AHPS_3 samples, indicating the uniform distribution of AHPS within the PAM matrix.

In‐situ radical polymerization (Figure 1a) yielded AHPS/PAM NC hydrogels featuring uniformly dispersed AHPS nanosheets. This network features dispersed AHPS nanosheets (4 µm × 10 µm × 1.6 nm; Figure S1) encapsulated and intertwined by PAM chains formed during acrylamide monomer polymerization. This intimate association establishes interfacial‐bridging, dynamic hydrogen bonding involving AHPS amine groups (–NH_2_), PAM amide groups (–CONH_2_), and water molecules—a reversible interaction motif critical for dissipative toughening under swollen conditions.

**FIGURE 2:**
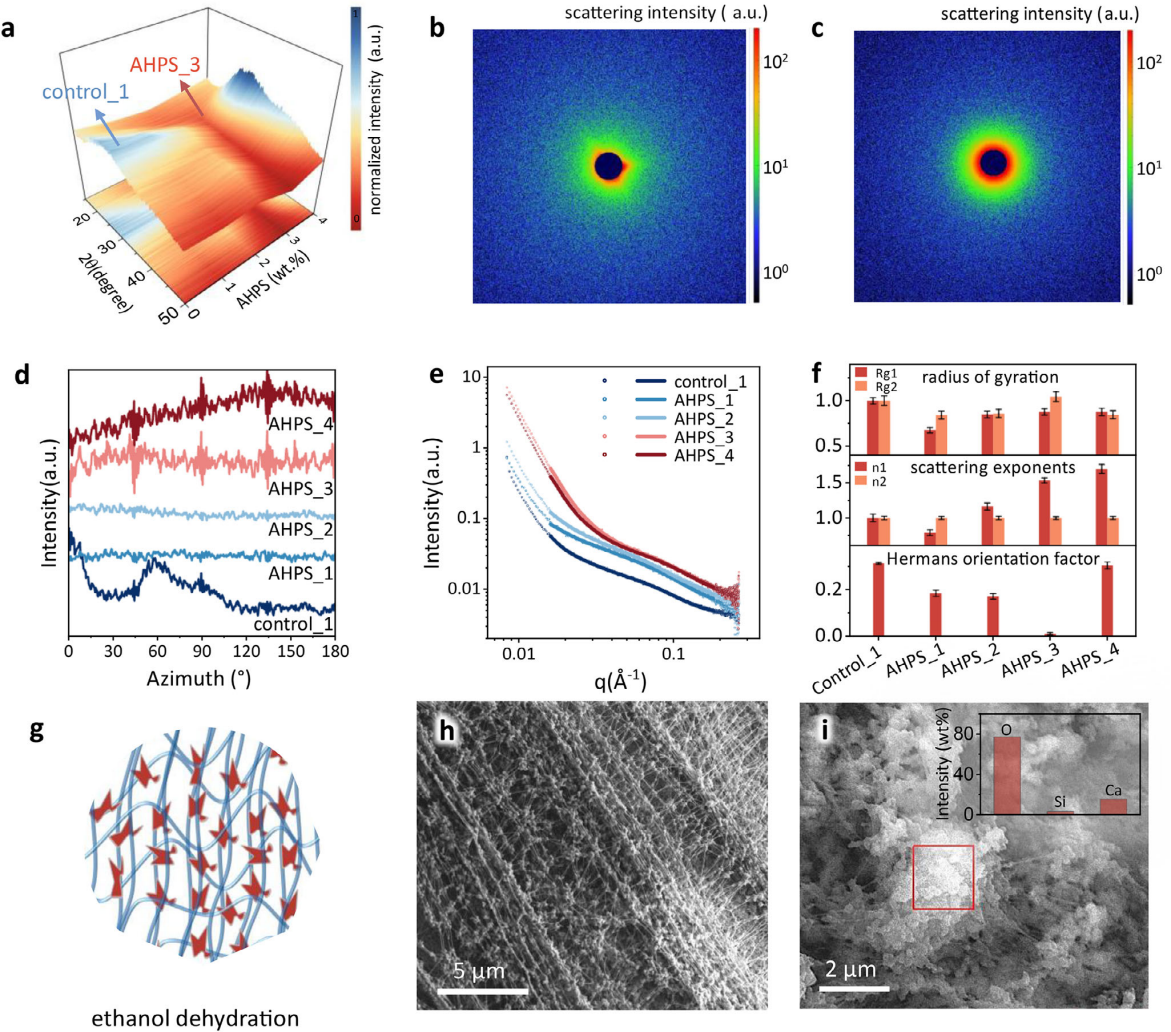
Multi‐scale structural analysis and network evolution. (a) XRD patterns of control_1 and AHPS/PAM NC hydrogels with AHPS dosages of 1–4 wt.%. (b,c) Representative 2D SAXS patterns for PAM and AHPS_3 samples, respectively. (d) Azimuthal intensity plots used to assess orientational order. (e) Azimuthally averaged SAXS profiles I(q) fitted using a two‑level Guinier–Porod (unified) model. The fits provide the radii of gyration *R*_*g*,1_ and *R*_*g*,2_ for the two structural levels and the associated scattering exponents *n*_1_ and *n*_2_. (f) Summary metrics extracted from SAXS: radius of gyration (*R*_*g*,1_, *R*_*g*,2_), scattering power‑law exponents (*n*_1_, *n*_2_), and the Herman orientation factor (*f_H_*) as a function of AHPS dosages. For clarity, the bar heights of *R_g_* and *n* are normalized to the PAM control (PAM = 1). Bars show means, error bars denote ±SD. The corresponding absolute values extracted from Unified Guinier–Porod fits are provided in Table S2. (g) Schematic of the AHP nodules (red) embedded in and interconnected by the PAM network (blue); ethanol dehydration route noted. (h,i) SEM images of critical‐point‐dried NC gel cross‐sections, highlighting the polymer–particle network. Insets: Elemental maps confirming Ca, Si, and O signals in the nodule‐like domains.

Scanning electron microscopy (SEM) images revealed a disordered 3D network in the pure PAM hydrogel (Figure 1b). In contrast, the AHPS/PAM NC hydrogel with 3 wt.% AHPS (AHPS_3) exhibited a densified cellular network, featuring an average pore size of ∼2 µm and robust pore walls (∼0.5 µm thick) (Figure 1c and Figure S2). This distinct structure arises from the uniform dispersion of AHPS nanosheets, which guide the polymerization and alignment of PAM chains. Elemental mapping within the AHPS/PAM nanolayers confirmed a uniform distribution of Ca (Figure 1e), corroborating the homogeneous dispersion of AHPS within the PAM matrix. To investigate the native network structure while minimizing ice‐templating artifacts, using ethanol gradient dehydration and critical‐point drying, AHPS nanosheets were readily observed within the PAM network (Figure 2g–i). AHPS domains appeared as nodule‐like structures bridged by polymer chains. These “nodules” are interpreted as AHPS nanosheets that have undergone contraction or curling during the solvent exchange and drying process, further confirming their distinct integration within the PAM network.

Wide‐angle X‐ray diffraction (XRD) and small‐angle X‐ray scattering (SAXS) were used to assess the multiscale structure of the swollen gels (Figure 2). XRD revealed variations in the characteristic peaks of PAM upon AHPS incorporation (Figure 2a and Figure S3), indicating the formation of a nanocomposite rather than a simple physical mixture. As the AHPS dosage increased from 1 to 3 wt.%, the characteristic PAM two broad amorphous halos centered at approximately 27° and 41° gradually diminished (Figure 2a and Figure S1), indicating an amorphous nanocomposite with no detectable crystalline aggregation within the XRD sensitivity. Subtle variations in the halo intensity/shape suggest changes in local chain packing and polymer‐filler interactions, while SAXS provides quantitative insight into nanoscale organization.

In swollen‐state 2D‐SAXS, PAM exhibits noticeable angular anisotropy (*f_H_* ≈0.31), which may arise from processing‐induced residual orientation and/or mesoscale density inhomogeneity and should not be interpreted as lamellar ordering. With increasing AHPS loading, the angular anisotropy is progressively reduced: *f_H_* decreases to 0.18 (AHPS_1) and 0.17 (AHPS_2), and reaches a near‐zero minimum at 3 wt.% AHPS (*f_H_* ≈0.01) (Figure 2b–d,f and Figure S4 and Table S2), indicating a quasi‐isotropic network with no evidence of long‐range nanosheet alignment. At 4 wt.% AHPS, anisotropy reappears (*f_H_* ≈0.30), suggesting aggregation and/or packing constraints at high loading. Azimuthally averaged SAXS profiles *I*(*q*)were fitted using a two‐level Unified Guinier–Porod model (Figure 2e,f and Table S2). The largerscale radius of gyration *R*_*g*,1_shows a non‐monotonic dependence on AHPS loading: it decreases sharply at 1 wt.% (201.71 → 136.34 nm) and then increases/plateaus at 2–4 wt.% (∼171–177 nm), indicating a transition from suppressing the original large‐scale heterogeneity of PAM to establishing a new filler‐regulated mesoscale correlation length. Meanwhile, the corresponding powerlaw exponent *n*_1_increases substantially at higher loadings (e.g., 2.19 → 3.367 at 3 wt.%), consistent with progressively densified domains and sharper interfacial contrast. The smaller‐scale structural level *R*_*g*,2_remains in a relatively narrow range (∼16–20 nm), implying that AHPS mainly regulates mesoscale organization/domain connectivity rather than generating an externally aligned lamellar architecture. Collectively, these multiscale features identify 3 wt.% AHPS as an optimal loading that yields nearisotropic scattering (minimum *f_H_*) while maintaining optical transparency and moldability (Figure 1d).

### 2.2 Breaking the Toughness‐Stretchability Trade‐off

**FIGURE 3:**
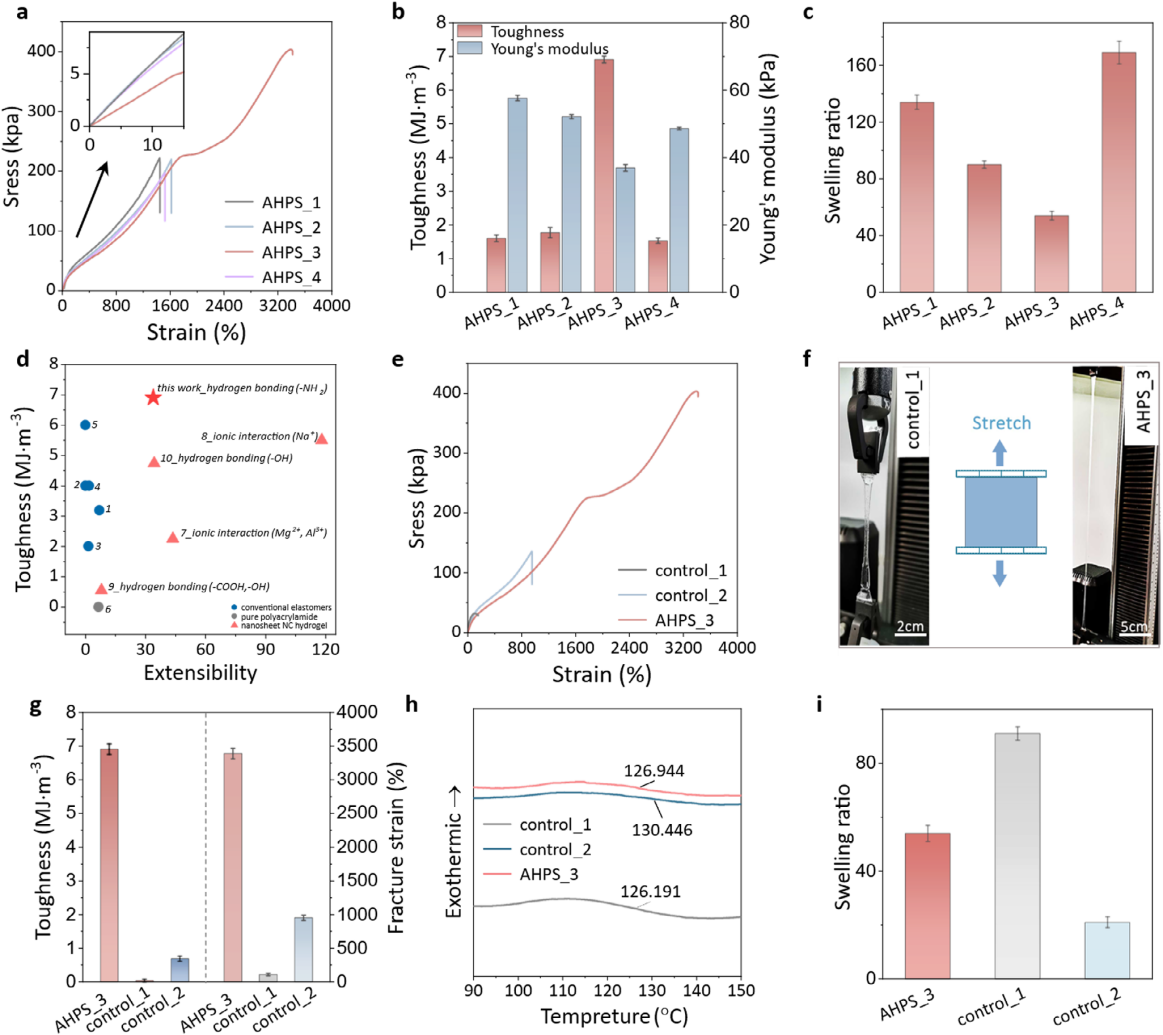
Mechanical and physical properties of AHPS/PAM NC hydrogels. (a) Tensile stress‐strain curves of AHPS/PAM NC hydrogels with AHPS dosage of 1–4 wt.% (AHPS_1‐4), revealing optimal mechanical performance at 3 wt.% AHPS. The inset shows the initial linear region (<15%) used to calculate Young's modulus. (b) Young's modulus and toughness (calculated from the stress‐strain curves) as functions of AHPS dosage. (c) Swelling ratios of AHPS/PAM NC hydrogels as a function of AHPS contents. (d) Ashby plot illustrating the toughness‐extensibility relationship of AHPS_3 compared to conventional elastomers [26, 27], pure PAM networks [28], and nanosheet NC hydrogels engineered with distinct non‐covalent interactions [29, 30, 31, 32], demonstrating Pareto‐optimal performance. (e) Tensile stress‐strain curves of pure PAM hydrogel (control_1), chemically crosslinked PAM hydrogel (control_2), and AHPS_3. (f) Photographs capturing tensile deformation of control_1 and AHPS_3 at different strain levels. (g) Comparison of toughness and fracture strain, showing a 173‐fold increase in toughness and a 31‐fold increase in elongation at break for AHPS_3 relative to control_1. (h) DSC curves, indicating a slight increase in glass transition temperature for AHPS_3. (i) Swelling ratio of control and AHPS_3.

The mechanical properties of AHPS/PAM NC hydrogels are strongly dependent on the AHPS dosage, with 3 wt.% AHPS identified as the optimal concentration for balancing between strength and stretchability (Figure 3a). Below this concentration, increasing AHPS progressively improves both tensile strength and elongation at break, yielding a high yield strength (410 kPa) and a fracture strain (3390%) at 3 wt.% AHPS. To quantify small‑strain stiffness, we determined the Young's modulus by linear fitting within the elastic regime (strain < 15%; Figure 3a, inset). Notably, AHPS‑1 exhibits the highest modulus despite its lowest nanosheet content and highest swelling ratio. Because strain < 15% primarily probes the small‑deformation response of the load‑bearing network, we attribute the high initial stiffness of AHPS_1 mainly to a higher effective density of elastically active chains in the chemically crosslinked PAM network (composition‑dependent crosslinking efficiency/network defects) [22, 23]. In contrast, at 3 wt.% AHPS, abundant reversible AHPS‐PAM associations provide additional relaxation/rearrangement pathways at small strains, slightly reducing the apparent modulus while underpinning the substantially improved energy dissipation and toughness at larger strains (Figure 3a,b).

Beyond the high strength and extensibility, the AHPS‑3 tensile curve displays two stress‑softening regimes, including a second softening at ultra‑high strains. Analysis of the tangent modulus reveals a distinct transition at approximately 1520% strain (Figure S5), marking the onset of this second softening stage. This is followed by a transient reduction in the modulus and a clear re‑hardening beyond 1900% strain, indicating that the network retains load‑bearing capacity after extensive rearrangement rather than failing catastrophically. We attribute the first softening stage to matrix disentanglement and the rupture/reformation of weak physical interactions common to all compositions [4, 24, 25]. In contrast, the second stage is consistent with interfacial rearrangement and chain sliding within densified AHPS‐PAM domains, which develop only at the optimized 3 wt.% composition.

Consistent with these curve features, the overall improvement at 3 wt.% is attributed to effective reinforcement of the PAM matrix by well‑dispersed AHPS nanosheets and to dynamic hydrogen‑bonding interactions that facilitate energy dissipation during deformation. The layered structure of AHPS, coupled with its uniform dispersion, promotes efficient load transfer and stress distribution throughout the PAM matrix. However, exceeding the 3 wt.% AHPS threshold results in a marked decrease in mechanical performance. This decline is attributed to the aggregation of AHPS nanosheets, which disrupts the uniform stress distribution and creates a heterogeneous gel structure with compromised network integrity. Above 4 wt.% AHPS dosage, the material's significantly increased Yong's modulus hinders reliable mechanical testing, indicating a transition to a less‐ordered, more aggregated state (Figure 3b). This is further supported by the swelling behavior (Figure 3c) that the AHPS_4 NC hydrogel exhibits a higher swelling ratio than the AHPS_3 NC hydrogel. This structural change not only reduces mechanical performance but also underscores the crucial role of optimal AHPS dispersion in maintaining the material's beneficial layered microstructure.

At the optimal 3 wt.% AHPS dosage, the hydrogel exhibits a remarkable fracture toughness of 6.91 MJ/m^3^, exceeding values reported for many other soft materials. The Ashby plot (Figure 3d) highlights the superior combination of toughness and extensibility of the AHPS/PAM NC hydrogel compared to conventional elastomers, other nanosheet‐based nanocomposites, and systems relying on weaker physical interactions. This exceptional performance makes the material promising for applications requiring both high toughness and high flexibility.

The enhanced mechanical properties are directly linked to the homogeneity and layered microstructure and strong interfacial interactions between AHPS and PAM. Compared to the pure PAM hydrogel (control_1), the AHPS_3 NC hydrogel shows a substantially higher toughness and elongation at break (Figure 3e–g), representing a 173‐fold increase in toughness and a 31‐fold increase in elongation over pristine PAM. The homogeneous dispersion of AHPS nanosheets within the PAM matrix ensures efficient load transfer, while the dynamic hydrogen bonding network dissipates energy through the reversible rupture and reformation of hydrogen bonds. Differential scanning calorimetry (DSC) shows only a slight increase in the glass transition temperature (0.753°C) for the AHPS_3 NC hydrogel compared to pure PAM (Figure 3h), suggesting that the AHPS‐PAM interactions are primarily governed by hydrogen bonding and van der Waals forces, rather than strong covalent bonds. This is further supported by the swelling behavior (Figure 3i): the AHPS/PAM NC hydrogel exhibits a lower swelling ratio than pure PAM (control_1) but a higher swelling ratio than a chemically crosslinked PAM hydrogel (control_2). This indicates a balanced crosslinking density, contributing to the hydrogel's high toughness and stretchability.

### 2.3 Dynamic Response to Cyclic Loading

**FIGURE 4:**
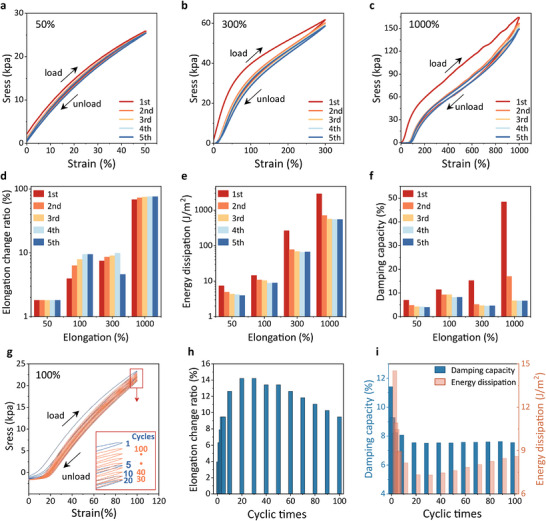
Cyclic tensile properties of AHPS/PAM NC hydrogel with 3 wt.% AHPS (AHPS_3). (a–c) Cyclic stress‐strain curves under repeated tensile loading to 50%, 300%, and 1000% strains for five cycles. (d–f) Quantification of elongation change ratio, energy dissipation, and damping capacity for five cycles at different strains. (g) Cyclic stress‐strain curves under repeated tensile loading to 100% strain for 100 cycles. (h,i) Evolution of elongation change ratio and energy dissipation and damping capacity as a function of cycle number at 100% strain.

The cyclic tensile behavior of AHPS_3 reveals a strain‐dependent mechanical response, characterized by a nonlinear relationship between energy dissipation and damping capacity (Figure 4a–f). At low strain (50%), the narrow hysteresis loops and minimal elongation change ratio suggest predominantly reversible deformation (Figure 4a,d), driven by the dynamic breaking and reforming of hydrogen bonds. However, as the strain increases to 300% and 1000%, the hysteresis loops widen significantly (Figure 4b,c), and the elongation change ratio rises dramatically, indicating substantial irreversible structural changes, such as chain scission and network rearrangement. This strain‐dependent behavior is further elucidated by the energy dissipation and damping capacity (Figure 4e,f). While the absolute energy dissipated increases with strain, the damping capacity decreases, reflecting a reduced efficiency of energy dissipation at higher strains. This apparent paradox arises from the irreversible structural damage induced by high strain, which disrupts the network's ability to dissipate energy effectively.

With 100 cycles of loading at a fixed 100% maximum strain, the material exhibits a two‐stage behavior, highlighting the dynamic equilibrium between irreversible damage and recovery mechanisms (Figure 4g–i). During the deformation stage (cycles 1–20), the increased loading cycles result in a increase in the elongation change ratio and a decrease in damping capacity and energy dissipation, indicating irreversible structural rearrangements, including hydrogen bond rupture and chain slippage. In the recovery stage (cycles 21–100), the elongation change ratio decreases with loading cycles while the energy dissipation gradually increases. This self‐recovery behavior explains the sudden reduction in the elongation change ratio during the fifth cycle at 300% strain (Figure 4d). Crucially, we performed strict controls to identify the origin of this recovery. The specimen mass remained virtually unchanged before and after cycling (variation of 0.19%), ruling out dehydration‐induced stiffening (Figure S6a). Furthermore, the mechanical response exhibits a strong dependence on rest time (Figure S6b): increasing the rest period from 10 s to 10 min leads to a stiffness recovery from 35.1% to 98.2%, while the toughness (energy dissipation) recovers from 44.7% to 96.3%. This time‐dependent reversibility, combined with the lack of mass loss, serves as a macroscopic signature of the re‐association of dynamic physical interactions and chain rearrangement, distinct from irreversible network damage. The self‐recovery mechanisms are related to hydrogen bond reformation and chain rearrangement, which are elaborated in the following sections. This two‐stage behavior reveals a dynamic balance between ongoing, low‐level irreversible damage accumulation and competing reversible recovery processes, underscoring the material's potential for long‐term stability and self‐healing.

### 2.4 Hydrogen Bonding and Water‐Mediated Network Dynamics

**FIGURE 5:**
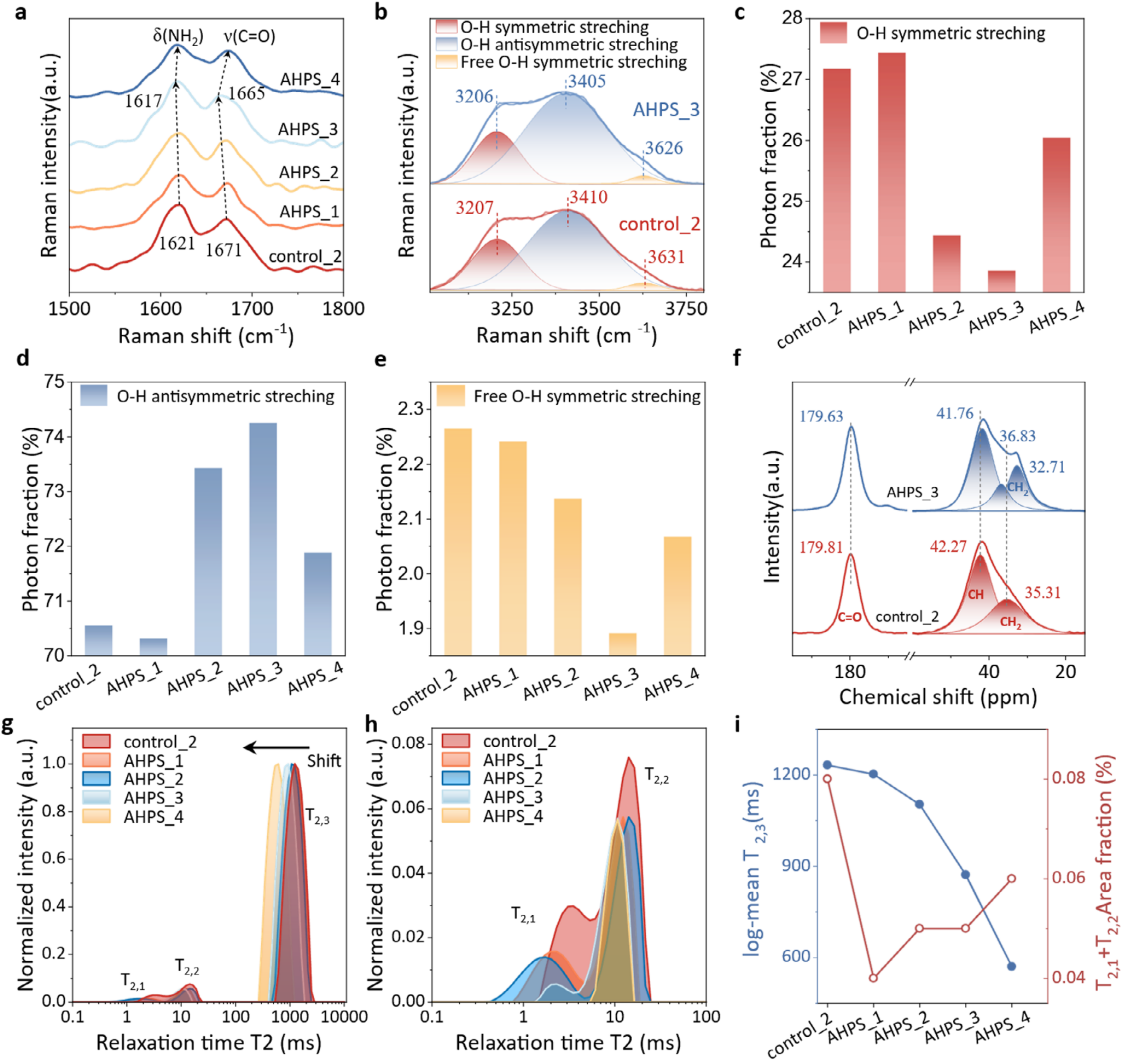
Dynamic hydrogen bonding network in AHPS/PAM NC hydrogels. (a) Raman spectra of chemically crosslinked PAM hydrogel (control_2) and AHPS/PAM NC hydrogels with 1–4 wt.% AHPS (AHPS_1‐4). (b) Raman spectra of control_2 and AHPS_3. The peaks are color‐coded to highlight the different O─H stretching vibrations. The free O─H symmetric stretching corresponds to O─H groups with weaker hydrogen bonds or transient “free” O─H bonds, closer to the symmetric stretching frequency in gas‐phase water. (c–e) The relative contributions of symmetric, antisymmetric, and free O─H symmetric stretching vibrations for control_2 and AHPS_1‐4 samples. (f) Solid‐state ^13^C MAS‐NMR spectra of control_2 and AHPS_3. (g) LF‐NMR T_2_ relaxation time distributions of the hydrogels, showing the dominant free water peak (T_2,3_) shifting toward shorter relaxation times with increasing AHPS content. Spectra were normalized by total area for each sample. (h) Magnified view of the short relaxation time region (0.1–100 ms), highlighting the minor populations of bound water (T_2,1_) and intermediate water (T_2,2_). (i) Variations in the log‐mean relaxation time of the free water component (T_2,3_, left axis) and the total area fraction of the restricted‐water components (T_2,1_+T_2,2_, right axis) across samples, with the restricted‐water fraction remaining at a negligible level (<0.1%).

The dynamic hydrogen bonding network in the AHPS/PAM NC hydrogel is a cornerstone of its exceptional mechanical properties. Unlike conventional nanocomposite hydrogels, which often rely on a single type of interaction, this system harnesses a complex synergy of hydrogen bonds between AHPS nanosheets, PAM chains, and water molecules. The formation of these hydrogen bonds, coupled with the active participation of water molecules, creates a robust and adaptable network that enhances both toughness and stretchability. The existence and functional significance of this network are compellingly demonstrated through Raman spectroscopy and solid‐state nuclear magnetic resonance (NMR) spectroscopy (Figure 5), which provides a molecular‐level understanding of how hydrogen bond formation and water molecule participation synergistically optimize the material's mechanical performance.

Raman spectroscopy reveals the formation and evolution of hydrogen bonds as a function of AHPS dosage (Figure 5a). As the AHPS dosage increases, the Raman shifts of the NH_2_ and C═O vibrational modes progressively decrease, reaching minima at AHPS_3. This systematic reduction in wavenumber signifies strengthened hydrogen bonding interactions, reflecting a more cohesive and ordered network. However, at AHPS_4, the relevant bands exhibit an abrupt blue shift, indicating a saturation or weakening of the interfacial hydrogen‑bond network. The weaker hydrogen bonding interactions in AHPS_4, indicated by the spectral blue shift, are attributed to a combination of structural restacking, hydration competition, and charge effects. First, SAXS analysis provides quantitative evidence of restacking: as shown in Figure 2f and Table S2, the Porod power‑law exponent (*n*) increases from ∼1.0 (AHPS_1) to ∼1.6 (AHPS_4). This transition from rod‐like to denser, sheet‐like structures suggests that restacking and site crowding reduce the accessibility of amine groups for multipoint binding. Second, hydration‐mediated competition plays a critical role: the higher swelling ratio of AHPS_4 (Figure 3c) indicates a thicker hydration shell, where water molecules compete as H‐bond donors/acceptors, partially disrupting amine‐silicate interactions. Finally, charge effects contribute to this behavior: the high concentration of AHPS introduces significant electrostatic repulsion within the network. This repulsion promotes network expansion (swelling), which spatially separates the polymer chains from the nanosheets and weakens the effective interaction density. Further insights into the hydrogen bonding environment are provided by the O‐H stretching modes of water molecules in the hydrogels (Figure 5b). The shift of all O‐H stretching modes to lower wavenumbers in AHPS_3, compared to control_2, suggests stronger hydrogen bonding of water molecules within the network. This restricted water mobility was independently verified by low‐field NMR (T2) relaxation analysis [33, 34], where the dominant water peak shifted toward shorter relaxation times with increasing AHPS content, confirming enhanced water confinement (Figure 5g–i and Table S3). In addition, the relative intensity of O‐H stretching modes shows that AHPS_3 contains the strongest antisymmetric stretching and the weakest symmetric stretching and free OH symmetric stretching (Figure 5c–e). This indicates the strongest hydrogen bonding network in AHPS_3, which yields its superior toughness, stretchability, and self‐recovery performance as shown in Figures 3 and 4.

Complementing the Raman spectroscopy results, solid‐state ^13^C NMR spectroscopy offers direct evidence for hydrogen bond formation (Figure 5f). The upfield shift of the carbonyl carbon (C═O) resonance in the PAM amide groups, coupled with enhanced peak intensity, directly evidences stronger hydrogen bonding between the AHPS amine groups and the PAM amide groups. Additionally, the upfield shifts of the PAM backbone carbons (CH and CH_2_) indicate the formation of new hydrogen bonds, extending the network beyond the amide groups to the polymer backbone.

### 2.5 Hierarchical Energy Dissipation

**FIGURE 6:**
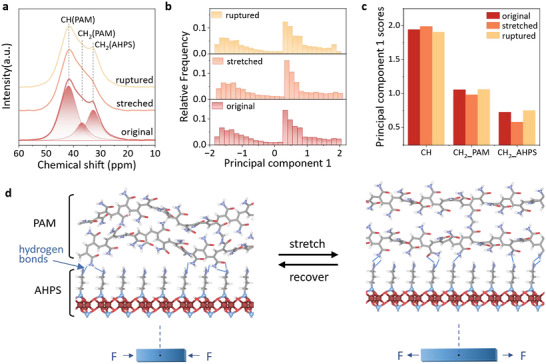
Deformation mechanisms of the AHPS/PAM NC hydrogels. (a) Solid‐state ^1^ ^3^C NMR spectra of AHPS_3 under different deformation states: original, stretched (with 1000% elongation rate), and ruptured. (b) Principal component 1 (PC1) loadings and their relative frequency from Principal Component Analysis (PCA) of the ^13^C CPMAS NMR spectra in (a). (c) PC1 scores for the CH, CH_2_ (PAM), and CH_2_ (AHPS) groups. (d) Schematic illustration of the deformation and recovery mechanisms of AHPS/PAM NC gels, emphasizing the roles of the layered AHPS structure, dynamic hydrogen bonding, and chain sliding.

The deformation mechanism of the AHPS/PAM NC hydrogel at the molecular level was elucidated through solid‐state NMR spectroscopy under varying strain conditions, capturing a dynamic view of structural transformations (Figure 6). The ^13^C NMR spectra of AHPS_3 were acquired in three distinct states: original (undeformed), stretched (with 1000% elongation rate), and ruptured (Figure 6a). Principal component analysis (PCA, Figure 6b) of these spectra revealed significant conformational changes in both the PAM chains and AHPS nanosheets during deformation.

The decrease in the CH_2_ (AHPS) score during stretching (Figure 6c) points to enhanced mobility and alignment of the AHPS nanosheets along the strain direction, indicative of nanosheet sliding. This movement is facilitated by the dynamic rupture and reformation of hydrogen bonds. Simultaneously, the PAM chains exhibit a slight reduction in mobility and a degree of alignment, as suggested by the minor decrease in the CH_2_ (PAM) score, likely due to their interaction with the sliding nanosheets. Upon rupture, both CH_2_ (PAM) and CH_2_ (AHPS) scores return to near‐normal values, signaling the loss of strain‐induced alignment and a reversion to a disordered state, consistent with material failure. The fluctuation in the CH score, likely linked to the polymer backbone, hints at a more intricate response involving both conformational and dynamic changes.

Building on these spectroscopic insights and the observed macroscopic behavior, the following deformation and recovery mechanisms are proposed (Figure 6d): The homogeneity and layered structure of the AHPS/PAM NC hydrogel, coupled with its dynamic hydrogen bonding network, enables highly efficient energy dissipation during deformation. Within elastic strain range, hydrogen bonds between the AHPS nanosheets and PAM chains dynamically rupture and reform, allowing nanosheets to slide relative to one another. This sliding process dissipates substantial energy, contributing to the AHPS/PAM NC hydrogel's exceptional toughness, stretchability, and self‐recovery performance. When the applied strain surpasses the capacity of this mechanism, irreversible chain scission or disentanglement of PAM chains from the nanosheets occurs, leading to material failure. The return of the CH_2_ scores to near‐normal values supports this, indicating the collapse of the ordered structure formed during stretching. Despite the rupture of individual hydrogen bonds and polymer chains, the AHPS/PAM NC hydrogel's layered structure and residual interaction network facilitate significant energy dissipation prior to complete failure, resulting in a ductile fracture behavior rather than brittleness.

## 3 Discussion

The exceptional mechanical performance of the AHPS/PAM NC hydrogel arises from a hierarchical energy‐dissipation process enabled by the interplay between a densified, quasi‐isotropic AHPS network and a dynamic hydrogen‐bonding network. This study provides a molecular‐level blueprint for overcoming the traditional trade‐off between toughness and stretchability. Crucially, our combined structural and mechanical analyses allow us to distinguish the roles of the nanofiller skeleton and dynamic interfacial interactions. The 2D‐SAXS results reveal a Hermans orientation factor close to zero (*f*_H_ ≈ 0.01), confirming that the AHPS nanosheets are dispersed as a densified, isotropic physical network rather than an oriented layered scaffold. We attribute the increase in initial stiffness to the static load‐bearing and constraint effects of this isotropic nanosheet skeleton. Notably, however, the hydrogel's pronounced hysteresis and high toughness cannot be rationalized by static load sharing alone, which would be expected to yield limited autonomous recovery after large deformation. Instead, the rapid and near‐complete recovery of toughness (∼96.3% within 10 min, Figure S6), together with the reduction of residual strain, strongly supports a dominant contribution from reversible dissociation and reformation of interfacial hydrogen bonds. Spectroscopic evidence of structural evolution during deformation—specifically regarding the interaction between AHPS domains and PAM chains—further supports the importance of interfacial interactions in delaying failure. Collectively, these results suggest that the interconnected AHPS network helps suppress catastrophic crack propagation and promotes ductile rather than brittle fracture.

Our findings bridge fundamental mechanics and practical application. The robust self‐recovery under cyclic loading supports long‐term durability against fatigue in real‐world use. From a manufacturing perspective, the high aqueous dispersibility of AHPS enables a scalable, one‐pot in situ polymerization process, avoiding complex processing steps often required for nanocomposites. Moreover, the nanosheet domains provide a versatile platform for functionalization (e.g., incorporating conductive or stimuli‐responsive guests) [35, 36, 37], provided that the critical hydrogen‐bonding network remains effective. By elucidating the link between molecular dynamics and macroscopic resilience, this work advances the design of nanosheet‐reinforced hydrogels and establishes a scalable framework for next‐generation soft engineering materials.

## 4 Materials and Methods

The chemicals used for fabricating the nanocomposite hydrogels were mostly acquired from Aladdin Co., Ltd., China. Ultrapure water (resistivity ≥ 18.2 MΩ·cm) was obtained from a Milli‐Q water purification system. All chemicals were used as received without further purification.

### 4.1 Synthesis of AHPS Nanosheets

AHPS nanosheets were synthesized following a modified sol‐gel procedure based on a previously reported method [38, 39]. Briefly, (3‐Aminopropyl) triethoxysilane (ATES, 5.85 mmol, 1.30 mL) was added dropwise to a solution of CaCl_2_·2H_2_O (4.3 mmol, 0.63 g) in anhydrous ethanol (20 mL) under vigorous magnetic stirring at 25°C. An aqueous NaOH solution (4.0 mol/L) was then added dropwise to the mixture until the pH reached 13.5 ± 0.1, as monitored by a calibrated pH meter. This initiated the condensation reaction. The reaction mixture was stirred at 25°C for 24 h. The resulting white precipitate was collected by centrifugation (9000 rpm, 10 min) and washed three times with a mixture of ethanol and ultrapure water (volume ratio equals 1:1) to remove unreacted reagents and byproducts. The purified AHPS nanosheets were dried under vacuum at 40°C for 24 h to remove residual solvent.

### 4.2 Synthesis of AHPS/PAM NC Hydrogels

AHPS/PAM NC hydrogels were synthesized via in situ free‐radical polymerization. AHPS nanosheets were first dispersed in ultrapure water by ultrasonication at 150w for 15 min to obtain stable, homogeneous dispersions. Acrylamide (AM, 1.00 g, 14.1 mmol) and N,N’‐methylenebisacrylamide (MBAA, 7.0 mg, 0.045 mmol, 0.07 wt.% relative to AM) were added to the AHPS dispersion. The concentration of AHPS nanosheets in the dispersions was varied to achieve the desired AHPS/AM weight ratios in the final hydrogels (0, 1, 2, 3, and 4 wt.%). For a typical AHPS/PAM NC hydrogel synthesis with a 3 wt.% AHPS/AM ratio, 30 mg of dried AHPS nanosheets were dispersed in 10 mL of ultrapure water. The mixture was sonicated for 10 min in an ice bath (0 ∼ 4°C) to ensure complete dissolution of the monomers and homogeneous mixing. Subsequently, an aqueous solution of ammonium persulfate (APS, 33 mg in 1 mL ultrapure water, 0.14 mmol, 0.33 wt.% relative to AM) and N,N,N’,N’‐tetramethylethylenediamine (TEMED, 500 µL, 3.3 mmol) were added sequentially to the pre‐cooled monomer/AHPS solution as the initiator and accelerator, respectively. The mixture was quickly degassed under vacuum for 10 min to remove dissolved oxygen. The degassed solution was immediately poured into rectangular molds (dimensions: 2 × 10 × 50 mm^3^), sealed, and placed in a controlled‐temperature chamber at 25°C for 22 h to allow for complete polymerization. The resulting AHPS/PAM NC hydrogels had a water content of approximately 80 wt.%, as determined by gravimetric analysis after drying.

### 4.3 Material Characterization

The supporting information provide details regarding the structural, morphological, spectroscopic, and mechanical characterization of the AHPS/PAM NC hydrogels.

## Author Contributions

Y.G.: Conceptualization. Y.G.: Methodology. Y.G. and Y.T.: Investigation. Y.T. and F.W.: Supervision. Y.G. and Y.T.: Writing – original draft. Y.G., Y.T., R.P., and F.W.: Writing – review & editing.

## Conflicts of Interest

The authors declare no conflicts of interest.

## Supporting information




**Supporting File**: advs74844‐sup‐0001‐SuppMat.pdf.

## Data Availability

The data that support the findings of this study are available in the supplementary material of this article.
