# Supplementary material for: Breaking the Toughness‐Stretchability Trade‐Off in Hydrogels with Dynamic Hydrogen Bonding
Source: Adv Sci (Weinh). 2026 Mar 14;13(36):e22530. doi: 10.1002/advs.202522530 (PMC13317752; doi:10.1002/advs.202522530)
Supplement: Supplementary file 1 — Supporting File: advs74844‐sup‐0001‐SuppMat.pdf. [file ADVS-13-e22530-s001.pdf]

Supplementary Information for

Breaking the toughness-stretchability trade-off in hydrogels with dynamic hydrogen bonding

Yining Gao *et al.*

This PDF file includes:

Supplementary Notes
Figures S1 to S6
Tables S1 to S3

Supplementary Notes

Structural and Morphological Characterization

X-ray diffraction (XRD): XRD patterns were recorded on a Bruker AXS D8 Advance diffractometer using Cu Kα radiation (λ = 1.5418 Å) at 40 kV and 40 mA. Samples were scanned in the 2θ range of 3 ~ 60° with a step size of 0.02° and a scan rate of 2°/min.

small-angle X-ray scattering (SAXS): SAXS was performed on a Xeuss 3.0 using Cu Kα radiation (E = 8.05 keV, λ = 1.54189 Å) and an EIGER2 R 1M detector (75 μm pixels) at a sample-detector distance of 1000 mm under vacuum (<1 mbar); a ~0.9 × 0.9 mm^2 beam was used, and each sample (sealed with Kapton) was measured for a total of 1200 s, with blank-film backgrounds acquired under identical conditions; the accessible scattering-vector range was q = 0.008-0.10 $Å^{-1}$.

Azimuthal anisotropy: Azimuthal intensity profiles $I(\chi)$ were obtained by annular integration over the primary scattering ring in the 2D patterns (the annulus was centered at the primary peak in the radially averaged $I(\mathrm{q})$, while masking beamstop and detector artifact regions. Before orientation analysis, χ was folded to 0-180°. The azimuthal angle χ was sampled over 0-360° into 1024 bins (Δχ ≈ 0.352°), with the macroscopic alignment axis defined as χ = 0°. Standard background subtraction and masking were applied. The Herman orientation factor, denoted f_H, was computed as(*1*)

$$f_H = [3\langle\cos^2\varphi\rangle - 1]/2$$

$$\langle\cos^2\varphi\rangle = \int_0^{180} I(\chi)\cos^2\varphi\, d\chi / \int_0^{180} I(\chi) d\chi$$

where φ is the angle between the scattering vector and the alignment axis. Values were averaged over three specimens and multiple regions (mean ± SD).

Unified Guinier-Porod analysis (two-level model; reporting R_g and n): Radially averaged SAXS profiles $I(\mathrm{q})$ were fitted with a two-level unified Guinier-Porod model(*2*). Each structural level i is represented by a Guinier form at low q and a Porod-type power law at high q, smoothly joined at a crossover q_i^*. We report the parameters $R_{g,1}$, $R_{g,2}$, n_1, and n_2. The Equation as follows

(1) Single level i (piecewise form):

$$I_i(\mathrm{q}) = G_i e^{-q^2 R_{g,i}{}^2/3}, \text{ for } \mathrm{q} \le q_i^*$$

$$I_i(\mathrm{q}) = G_i e^{-n_i} \cdot (\frac{q_i^*}{q})^{n_i}, \text{ for } \mathrm{q} > q_i^*$$

(2) Crossover:

$$q_i^* = \sqrt{3n_i}/R_{g,i}$$

(3) Total two-level intensity.

$$I(\mathrm{q}) = I_1(\mathrm{q}) + I_2(\mathrm{q})$$

In Eqs. (1)-(3), $I(\mathrm{q})$ is the azimuthally averaged scattering intensity and q is the magnitude of the scattering vector. Each structural level i is modeled by a Guinier term at low q and a Porod type power law at high q, smoothly joined at the crossover q_i^* such that both the intensity and its first

derivative are continuous; G_i is a level specific scale factor determined by fitting (used to ensure continuity, not interpreted physically), $R_{g,i}$ is the radius of gyration, and n_i is the high q power-law exponent, with $q_i^* = \sqrt{3n_i}/R_{g,i}$. The total intensity is the sum of two levels, $I(\text{q}) = I_1(\text{q}) + I_2(\text{q})$. The reported model parameters are $\{R_{g,1}, R_{g,2}, n_1, n_2\}$. Fits were performed over the measured range q = 0.008-0.10 $Å^{-1}$, and parameter uncertainties were taken from the fit covariance matrix.

Scanning electron microscopy (SEM): The morphology of the hydrogels was examined using a FEI Quanta 450 FEG SEM operating at an accelerating voltage of 200 kV. Samples were cryo-fractured in liquid nitrogen, sputter-coated with gold, and imaged. The AHPS/PAM NC gel samples were prepared using two distinct drying methods: freeze-drying and supercritical CO_2 drying. For freeze-drying, the hydrogel sample was first rapidly quenched in liquid nitrogen to freeze the structure. The frozen sample was then carefully fractured to expose the internal morphology and placed in the cold trap of a freeze-dryer at - 60°C for vacuum freeze-drying. This method removes the water content while preserving the porous structure of the hydrogel. For supercritical CO_2 drying, the hydrogel sample underwent ethanol gradient dehydration to gradually replace the water within the network. The ethanol concentrations used were 50%, 60%, 70%, 75%, 80%, 85%, 90%, 95%, and 100%, with the sample sequentially immersed in each solution starting from the lowest concentration. After dehydration, the sample was transferred to a supercritical CO_2 dryer, where the ethanol was replaced with liquid CO_2, which was then converted to a supercritical state. This process removes the ethanol while maintaining the structural integrity of the hydrogel network, ensuring that the dehydrated sample retains its original morphology for accurate characterization.

Transmission electron microscopy (TEM): TEM images were obtained using a JEOL JEM-2100F TEM operating at an accelerating voltage of 200 kV. Dilute AHPS dispersions were drop-casted onto carbon-coated copper grids and allowed to dry before imaging.

Solid state ^{13}C Nuclear Magnetic Resonance (NMR): Solid-state ^{13}C cross-polarization magic angle spinning (CP/MAS) NMR spectra were acquired on a Bruker AVANCE III 400 MHz spectrometer operating at a ^{13}C Larmor frequency of 100.6 MHz. Samples were packed into 4 mm zirconia rotors and spun at a MAS rate of 5 kHz. A contact time of 1 ms and a recycle delay of 5 seconds were used.

low-field NMR (LF-NMR): LF-NMR T_2 was measured on a NIUMAG VTMR20-010V (0.50 T ± 0.03 T; 1H ≈ 21.3 MHz) using a 4 mm probe at 25 °C with a CPMG sequence. Echo trains were inverted to T_2 distributions using nonnegative least squares with Tikhonov regularization (alpha ≈ 0.10) on a 0.1 - 10000 ms grid; spectra were normalized to total area. Distributions were resolved into short ($T_{2,1}$), intermediate ($T_{2,2}$), and long ($T_{2,3}$) components; the log-mean T_2 of the dominant long-T_2 component and its area fraction are reported.

Raman spectroscopy: Raman spectra were obtained using a Renishaw Invia equipped with a 633 nm laser. Spectra were collected in the 200 - 4000 cm^{-1} range with a resolution of 3 cm^{-1}.

Differential scanning calorimetry (DSC): DSC was performed using a STA 449 F3 Simultaneous Thermal Analyzer (Netzsch, Germany). The measurements were conducted over a temperature range of 30°C to 250°C at a heating rate of 10°C/min under a nitrogen atmosphere (flow rate: 50 mL/min). Samples were placed in aluminum crucibles, and an empty crucible was used as a

reference. The DSC curves were analyzed to determine the glass transition temperature of the AHPS/PAM NC gel

Swelling Ratio

The swelling ratio of the AHPS/PAM nanocomposite hydrogels was determined by immersing dried hydrogel samples in ultrapure water at 25 °C for 21d. Three independent measurements were performed for each sample, and the error bars in subsequent analyses represent the standard deviation (mean ± SD) of the replicate tests. The weight of the swollen hydrogels was measured at regular intervals until equilibrium swelling was reached. The swelling ratio (SR) was calculated using the following equation:

$$SR = (Ws - Wd) / Wd$$

where Ws is the weight of the swollen hydrogel at equilibrium and Wd is the weight of the dried hydrogel.

Mechanical Testing

Mechanical properties were evaluated using an Instron 5967 universal testing machine equipped with a 50 N load cell. All tests were performed at room temperature (25 ± 2 °C). Three independent measurements were performed for each sample, and the error bars in subsequent analyses represent the standard deviation (mean ± SD) of the replicate tests.

Tensile tests: Uniaxial tensile tests were performed on rectangular hydrogel samples (50 mm × 10 mm × 2 mm) at a constant crosshead speed of 50 mm/min until fracture. The tensile strength, elongation at break, and Young's modulus were determined from the stress-strain curves. At least five samples were tested for each composition.

Cyclic tensile tests: Cyclic tensile tests were performed on rectangular hydrogel samples (50 mm × 10 mm × 2 mm) at a constant crosshead speed of 50 mm/min. Samples were loaded to various predetermined elongations (50%, 100%, 300%, and 1000%) and then unloaded to the initial position. Multiple loading-unloading cycles were performed for each sample.

Water-loss control: Wet mass was recorded immediately before and within 30 s after 5 loading–unloading cycles to 300% engineering strain at 50 mm/min (25 ± 2 °C) using an analytical balance (readability 0.1 mg). Samples were handled on Parafilm and kept in a sealed container to prevent evaporation. The relative mass change was calculated as the percentage difference between the post-cycling mass and the initial mass.

Rest-time recovery: After one cycle to 300% strain at 50 mm/min, samples rested under zero load in a sealed container for 10 s or 10 min and were reloaded to the same strain. From the stress-strain curves, we obtained the Young's modulus, residual strain, and toughness. Recovery ratios were determined by dividing the values from the reloading curve by those from the virgin loading curve.

Data Analysis

Young's modulus: The Young's modulus (initial tangent modulus, was calculated from the slope of the stress-strain curve in the linear elastic region (strain < 15%).

Toughness: The toughness of the hydrogels was calculated as the area under the stress-strain curve up to the point of fracture, representing the energy absorbed per unit volume before failure. This was determined by numerical integration of the stress-strain curve using OriginPro software.

Energy dissipation: Energy dissipation during cyclic loading was quantified by calculating the area enclosed within the hysteresis loop (the difference between the areas under the loading and unloading curves) for each cycle. This represents the energy dissipated per unit volume per cycle.

Damping capacity: Damping capacity, often represented by the loss factor, a qualitative assessment of damping behavior can be inferred from the hysteresis loops in the cyclic tensile tests. Larger hysteresis loops generally indicate higher energy dissipation and, therefore, greater damping capacity.

Principal component analysis (PCA): PCA was performed on the ^{13}C CPMAS NMR spectra of the AHPS/PAM hydrogels to investigate the relationship between the hydrogel composition and the chemical environment of the different carbon groups. The spectral data were preprocessed by baseline correction and normalization. PCA was carried out using the MATLAB software (version 9.1. Principal component 1 (PC1) was found to capture the majority of the variance in the data (70% variance explained). Analysis of the PC1 loadings revealed significant contributions from the CH, CH_2 (PAM), and CH_2 (AHPS) groups, indicating that PC1 effectively differentiates between the PAM and AHPS components within the hydrogel network.

AHPS nanosheets morphology and structure

The observed XRD peaks, including (001), (130, 200), and (060, 330), align with the highly ordered layered structure of tri-octahedral talc (JCPDS card 13-0558, Figure S1a), confirming the structural integrity of AHPS post-incorporation. The sharpening of these peaks, particularly (130, 200) and (060, 330), indicates enhanced crystallinity and ordering within the AHPS nanosheets. Analysis reveals a Ca-Ca distance of 0.35 nm in the octahedral sheets, closely matching the value in calcium hydroxide ($Ca(OH)_2$, 0.359 nm)(*3*), suggesting a well-defined octahedral Ca arrangement.

Supplementary Figures

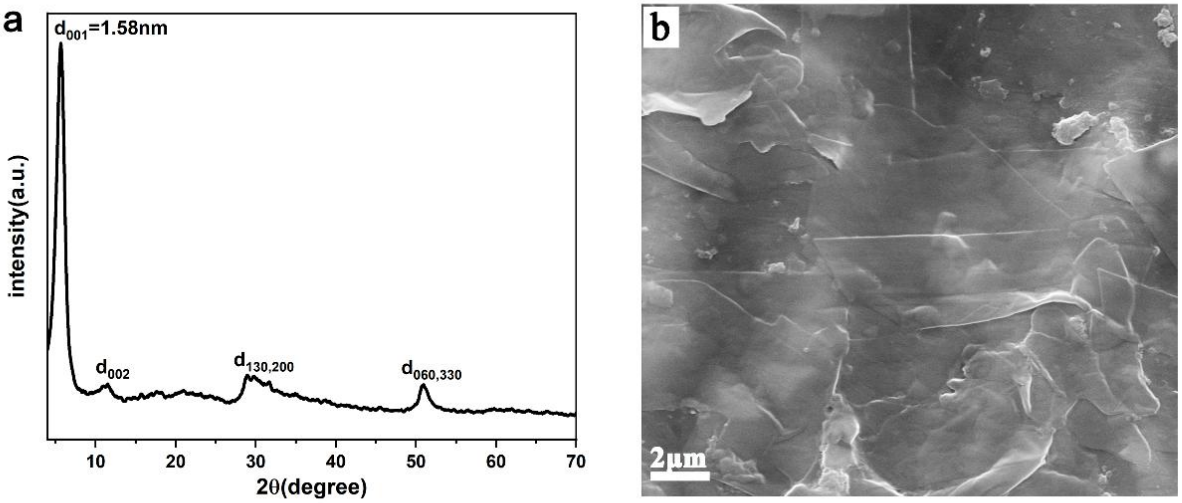

Figure S1. (a)XRD (b)SEM images for AHPS nanosheets morphology

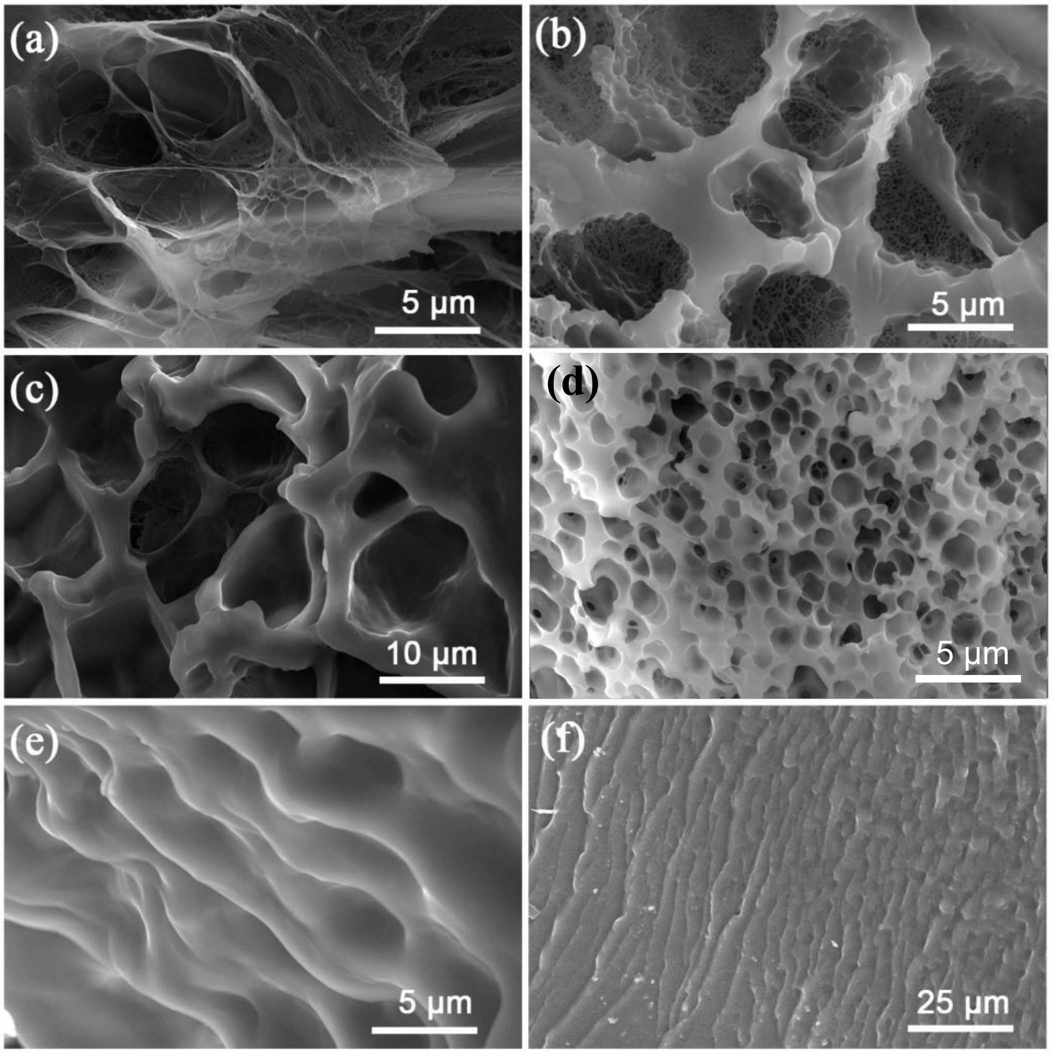

Figure S2. SEM images of AHPS/PAM NC gels showing the effect of AHPS dosage on microstructure. (a) 0 wt% AHPS (pure PAM), (b) 1 wt% AHPS, (c) 2 wt% AHPS, (d) 3 wt% AHPS, (e) 4 wt% AHPS, and (f) 3 wt% AHPS (dehydrated structure, showing the collapsed network)

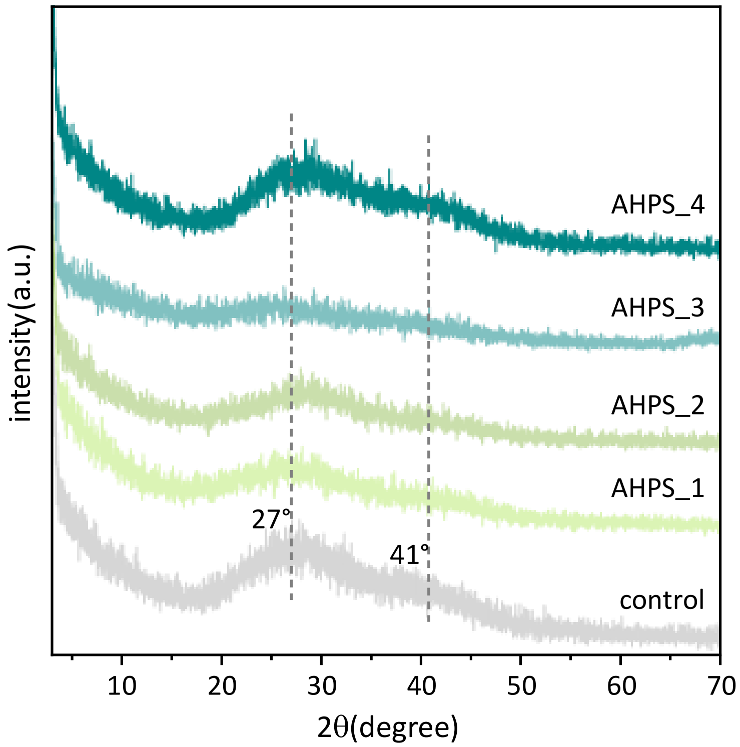

Figure S3. XRD patterns of the pure PAM control and AHPS/PAM NC gels with 1-4 wt AHPS (AHPS_1-4)

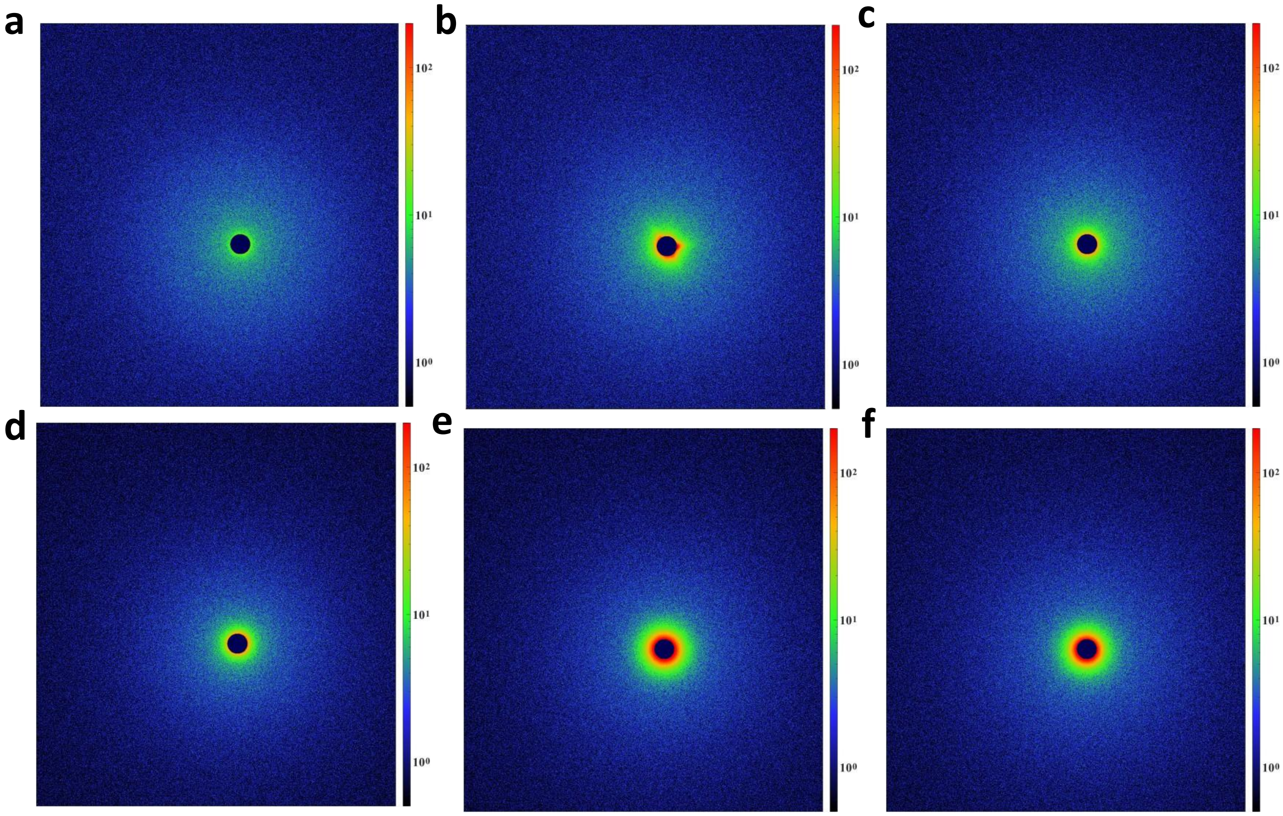

Figure S4. 2D SAXS patterns of (a) a blank Kapton film, (b) the pure PAM control, and (c-f) AHPS/PAM NC gels with AHPS contents ranging from 1 to 4 wt%

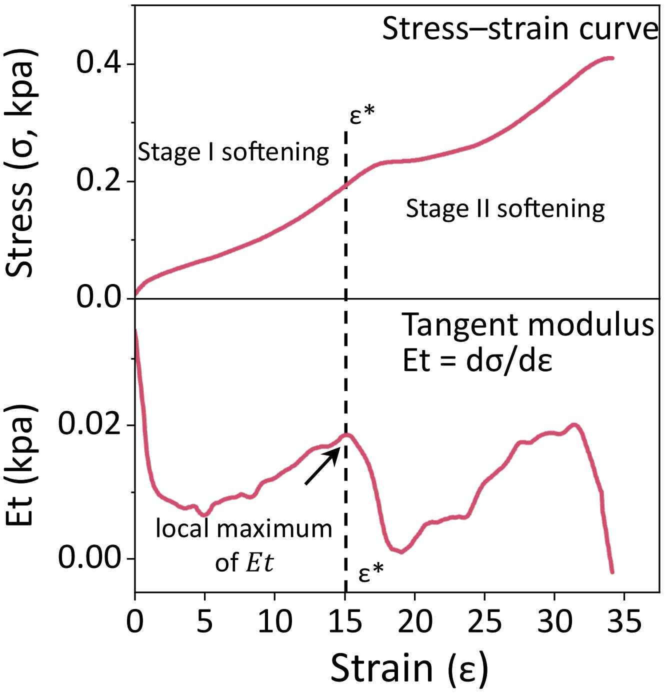

Figure S5. The engineering stress–strain curve was digitized from Fig. 3a AHPS_3 using Engauge and processed in Origin. The digitized data were converted to engineering strain ε (dimensionless) and resampled on a uniform $\Delta\varepsilon = 0.05$ grid via linear interpolation. The tangent modulus was computed as the numerical derivative $Et(\varepsilon) = d\sigma/d\varepsilon$ using a central-difference scheme. The Stage I and Stage II softening regions are associated with dips (local minima) in Et(ε), consistent with the plateau-like segment in the σ–ε curve. The vertical dashed line marks the transition strain ε*(15.2), defined as the last local maximum of Et(ε) immediately preceding the Stage II softening dip.

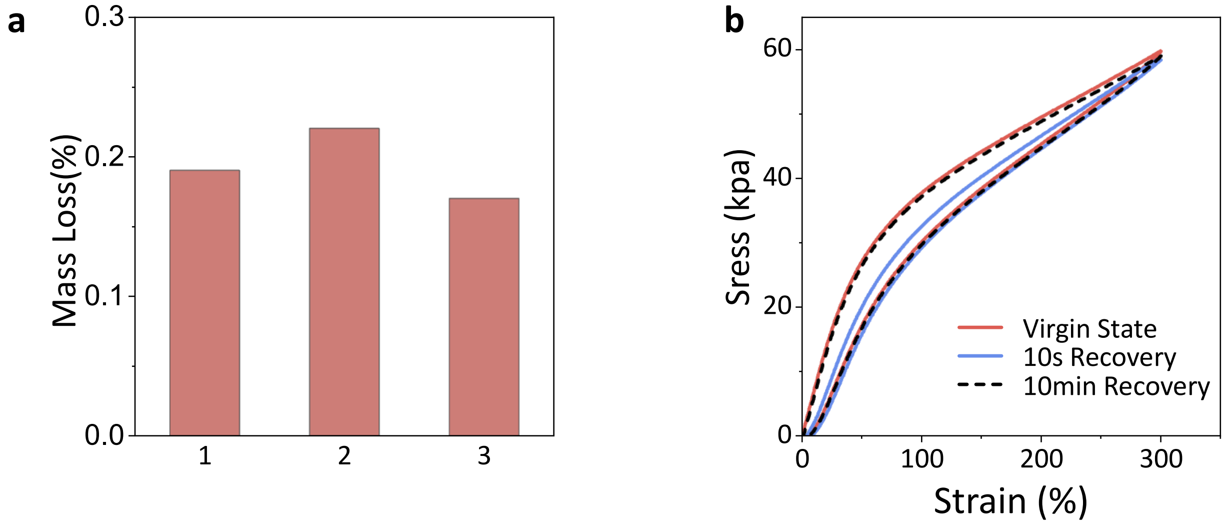

Figure S6. Rest-time-dependent mechanical recovery of the hydrogel after cyclic tensile loading. a) Mass change measured immediately before and after cyclic tensile tests (5 cycles to 300% strain), showing negligible mass loss. (b) Stress–strain curves of the 1st cycle (pristine), the 2nd cycle (softened), and subsequent reloading after resting for 10 s and 10 min. A longer resting time leads to a pronounced upward shift of the reloading curve and reduced residual strain, indicating partial recovery of the mechanical response after cyclic softening.

Supplementary Tables

Table S1. Mechanical property data used to construct the Ashby plot in Figure 2d. Includes data for AHPS/PAM hydrogels and other materials.

NO.	materials	extensibility [%]	Tensile strength [kPa]	Toughness [$MJ\cdot m^{-3}$]	Energy dissipation
1	nature rubber	800	700	3.19	conventional elastomers
2	bone	160000	3	4	conventional elastomers
3	elastin	2000	150	2	conventional elastomers
4	resilin	3000	190	4	conventional elastomers
5	high-tensile steel	1500000	0.8	6	conventional elastomers
6	polyacrylamide	11	660	0.002	polyacrylamide
7	layered double hydroxide/polyacrylamide	45.8	4361	2.25	($Mg^{2+,}$ $Al3^{+}$) ionic interaction
8	montmorillonite/polyacrylamide	100	11800	5.5	(Na^{+}) ionic interaction
9	laponite/graphene/cellulose nanofibrils/polyacrylamide	120	800	0.55	(-COOH, -OH) hydrogen bonding
10	graphene oxide/polyacrylamide	385	3435	4.74	(-OH) hydrogen bonding
11	AHPS/PAM hydrogels (this work)	3390	410	6.91	(-NH2) hydrogen bonding

Table S2. Summary metrics extracted from SAXS: radius of gyration ($R_{g,1}$, $R_{g,2}$), scattering power-law exponents (n_1, n_2), and the Herman orientation factor (f_H) as a function of AHPS dosages. Values in Figure 2f are normalized to PAM (PAM = 1)

Sample	$R_{g,1}$(nm)	$R_{g,2}$(nm)	n_1	n_2	f_H
Control_1	201.71 ± 7.30	19.06 ± 0.95	2.19 ± 0.12	4.00 ± 0.10	0.314 ± 0.004
AHPS_1	136.34 ± 6.40	16.06 ± 0.82	1.73 ± 0.09	4.00 ± 0.09	0.185 ± 0.013
AHPS_2	171.42 ± 7.57	16.39 ± 0.98	2.56 ± 0.11	4.00 ± 0.10	0.171± 0.012
AHPS_3	176.99 ± 7.08	19.92 ± 0.93	3.38 ± 0.08	4.00 ± 0.08	0.011 ± 0.005
AHPS_4	176.93 ± 8.10	16.13 ± 0.69	3.73 ± 0.14	4.00 ± 0.09	0.305 ± 0.015

Table S3. Summary of LF-NMR relaxation parameters for PAM (Control) and AHPS hydrogels. Parameters are reported as $T_{2,1}$ (short), $T_{2,2}$ (intermediate), and $T_{2,3}$ (long)

Sample	$T_{2,1}$+$T_{2,2}$ Area fraction [%]	$T_{2,3}$ Area fraction [%]
Control_2	0.08	99.92
AHPS_1	0.04	99.96
AHPS_2	0.05	99.95
AHPS_3	0.05	99.95
AHPS_4	0.06	99.94

References

1. L. E. Alexander, X-ray diffraction methods in polymer science. *John Wiley & Sons,Inc*, (1969).
2. G. Beaucage, Approximations leading to a unified exponential/power-law approach to small-angle scattering. *Applied Crystallography* **28**, 717–728 (1995).

3. J. Minet *et al.*, New layered calcium organosilicate hybrids with covalently linked organic functionalities. *CHEMISTRY OF MATERIALS* **16**, 3955–3962 (2004).
